# Abundance and Diversity of Ammonia-Oxidizing Archaea and Bacteria in Sediments of Trophic End Members of the Laurentian Great Lakes, Erie and Superior

**DOI:** 10.1371/journal.pone.0097068

**Published:** 2014-05-12

**Authors:** Annette Bollmann, George S. Bullerjahn, Robert Michael McKay

**Affiliations:** 1 Department of Microbiology, Miami University, Oxford, Ohio, United States of America; 2 Department of Biological Sciences, Bowling Green State University, Bowling Green, Ohio, United States of America; Catalan Institute for Water Research (ICRA), Spain

## Abstract

Ammonia oxidation is the first step of nitrification carried out by ammonia-oxidizing Archaea (AOA) and Bacteria (AOB). Lake Superior and Erie are part of the Great Lakes system differing in trophic status with Lake Superior being oligotrophic and Lake Erie meso- to eutrophic. Sediment samples were collected from both lakes and used to characterize abundance and diversity of AOA and AOB based on the ammonia monooxygenase (*amoA*) gene. Diversity was accessed by a pyro-sequencing approach and the obtained sequences were used to determine the phylogeny and alpha and beta diversity of the AOA and AOB populations. In Lake Erie copy numbers of bacterial *amoA* genes were in the same order of magnitude or even higher than the copy numbers of the archaeal *amoA* genes, while in Lake Superior up to 4 orders of magnitude more archaeal than bacterial *amoA* copies were detected. The AOB detected in the samples from Lake Erie belonged to AOB that are frequently detected in freshwater. Differences were detected between the phylogenetic affiliations of the AOA from the two lakes. Most sequences detected in Lake Erie clustered in the *Nitrososphaera* cluster (Thaumarchaeal soil group I.1b) where as most of the sequences in Lake Superior were found in the *Nitrosopumilus* cluster (Thaumarchaeal marine group I.1a) and the *Nitrosotalea* cluster. Pearson correlations and canonical correspondence analysis (CCA) showed that the differences in abundance and diversity of AOA are very likely related to the sampling location and thereby to the different trophic states of the lakes.

## Introduction

Ammonia oxidation is the first and rate-limiting step in nitrification, the oxidation of ammonia to nitrate via nitrite. Understanding this process and its controls is of high importance because it controls the availability of two major nitrogen compounds (ammonium and nitrate) in nature. The long-known Ammonia-oxidizing Bacteria (AOB) and the recently discovered Ammonia-oxidizing Archaea (AOA) use the oxidation of ammonia to nitrite as an energy-generating step[1], [2]. Since both groups use the same energy substrate it is important to understand the environmental conditions under which AOA or AOB dominate. Among the factors reported to influence the abundance and diversity of AOA and AOB are fertilizers (ammonium addition) [3], [4]; pH [5], [6]; salinity [7], [8] and oxygen [9]. For example AOA have much higher affinity for ammonium/ammonia than AOB [10]–[13] and are often detected in more oligotrophic environments like the open ocean or oligotrophic lakes [14]. In contrast AOB grow with higher rates in soils [1]–[4] and enrichment cultures [3], [4], [15].

AOB comprise a phylogenetically distinct group in the phylum *Beta-Proteobacteria* as well as a few marine strains in the *Gamma-Proteobacteria* [5], [6], . The betaproteobacterial AOB cluster in different groups based on environmental characteristics such as high- and low ammonium availability, salinity and pH [7], [8], [16], [17]. AOB found in freshwater systems generally belong to the *Nitrosomonas oligotropha*, *Nitrosomonas communis* and the *Nitrosospira* clusters [9], [18]–[21].

Recently the phylum *Thaumarchaeota* was described as a new deep-branching phylum in the archaeal domain [10]–[13], [22]–[24]. Besides groups of microorganisms with unknown physiology such as the groups pSL12 from hot springs, ALOHA from the open ocean and I.1c from acidic soils, AOA are a large group within the *Thaumarchaeota* [14], [25]. The AOA have been split into four groups: *Nitrosopumilus* (Thaumarchaeal marine group I.1a), *Nitrososphaera* (Thaumarchaeal soil group I.1b), *Nitrosotalea* (SAGMGC-1, formerly group I.1a associated) and *Nitrosocaldus* cluster (formerly, ThAOA group). Representatives of the *Nitrosopumilus* cluster have mainly been detected in aquatic marine and freshwater systems, *Nitrososphaera* cluster in soils and sediments, *Nitrosotalea* cluster in acidic soils and oligotrophic freshwater systems and *Nitrosocaldus* in extreme environments like hot springs [22], [26]. However, it has been shown that not all *amoA* encoding Thaumarchaeota are autotrophic ammonia oxidizers. Some exhibit a mixotrophic physiology, while others express the *amoA* gene but don't oxidize ammonium [27], [28].

Overall AOA communities in marine and soil environments are much better studied than the AOA in freshwater systems. Molecular surveys have been conducted to analyze AOA and/or AOB communities and the factors that control them in freshwater systems. Trophic status and ammonium availability are among the factors that influence the abundance as well as the structure of the AOA and AOB communities [7], [14], [18]–[21], [29]–[38].

The Laurentian Great Lakes system is the largest system of freshwater lakes on earth and is located in the eastern part of North America forming part of the border between the United States and Canada. Lake Superior, the largest and deepest of the five lakes, is mainly surrounded by forest and coincident with low human population abundance in the watershed, is least affected by pollution. At the opposite end of a trophic continuum is Lake Erie, the shallowest of the Great Lakes. With high population abundance and a watershed allocated largely to agricultural and industrial activities, Lake Erie is heavily impacted by urban and agricultural [39] runoff from the areas surrounding the lake and ranges from mesotrophic to eutrophic. In stark contrast, Lake Superior, which serves as the headwaters for the Great Lakes system, has remained pristine and is characterized as oligotrophic [40]. This is supported by historical data showing mainly flat profiles of total dissolved solids as well as concentrations of major ions which serve as indicators of anthropogenic impacts on the system [40], [41].

Seemingly counter to the static trends in major ions is the observation that Lake Superior has exhibited a continuous, century-long five-fold increase in nitrate levels from 5 µmol/l to 26 µmol/l [42]. Nitrification rates in the water column of Lake Superior are lower than in other freshwater systems including measurements in other parts of the lakes, but higher than in the open ocean – another indication that Lake Superior is an oligotrophic system [43], [44].

Here we present a study investigating the abundance and diversity of Ammonia-oxidizing Archaea and Bacteria in the sediments of western Lake Superior and embayments of western Lake Erie using a deep sequencing approach (454 pyrosequencing with barcoded primers). We chose samples from these two different sections of the Great Lakes, because they represent trophic end members of the Great Lakes system with Lake Superior being very oligotrophic and Lake Erie being meso/eutrophic. The results will give an insight into the phylogeny and distribution of AOA and AOB in the Great lakes and on the impact of the trophic state of freshwater environments on both groups of organisms.

## Materials and Methods

### Sediment sampling

**Figure 1:**
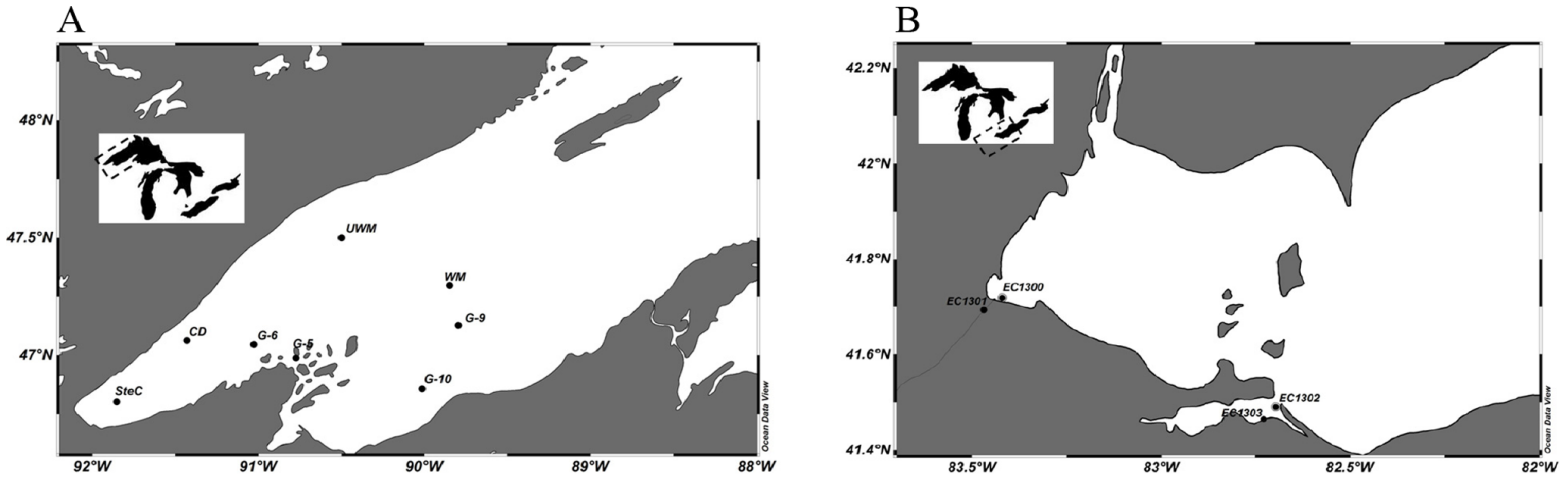
Sampling sites for Lake Superior (A) and Lake Erie (B) sediments.

Sediment samples were taken at Lake Superior and Erie stations (Fig. 1) in October 2010, maintained at 4°C and transported within 4 days after sampling to the laboratory at Miami University where they were frozen at −20°C upon arrival. The samples were taken in US territorial waters of the Great Lakes where no permissions are required as overseen by the International Joint Commission. No endangered species are involved. Two sampling techniques focusing on the top 0–10 cm of the sediment were utilized: ponar dredge (0-max. 10 cm) and sediment cores (0–5 cm). All Lake Erie samples (EC1300, EC1301, EC1302, EC1303) were taken with the ponar dredge (Table 1). At four process stations (CD, SteC, UWM, WM) in Lake Superior (Table 1), sediment cores were collected using an Ocean Instruments (San Diego, CA) MC-400 multi-corer. The other four Lake Superior samples were taken with the ponar dredge (Grab5, Grab6, Grab9, Grab10) (Table 1). Subsamples were taken from the mixed sediment material for determination of mineral nitrogen (ammonium, nitrite, and nitrate), dry weight and molecular analysis in triplicate.

**Table 1:** Environmental data from the sediment samples and the overlaying water.

	Lake	Sampling Date	Depth [m]	Distance to shore [km]	Sample	NH_4_^+^ 2) [µg N/g dw]	NO_3_^−^ 2) [µg N/g dw]	Chl *a* 1)[µg Chl *a*/l]	DO [mg/l] 3)
EC1300	Erie	10/13/10	9.6	Near shore	Ponar;	149.8±2.7	0.4±0.0	4.4±1.9	7.2
EC1301	Erie	10/13/10	8.3	River mouth	Ponar;	202.2±6.4	1.2±0.0	17.9±7.7	5.5
EC1302	Erie	10/13/10	12.6	Bay mouth	Ponar;	7.4±0.2	0.5±0.1	13.6±1.3	10.0
EC1303	Erie	10/13/10	7.2	Near shore	Ponar;	59.5±2.5	0.5±0.0	20.2±1.1	nd
									
Grab5	Superior	10/07/10	30	Near shore	Ponar	0.1±0.1	1.3±0.2	nd	nd
Grab6	Superior	10/07/10	30	Near shore	Ponar	1.3±0.9	2.6±0.5	nd	nd
Grab9	Superior	10/06/10	nd	28.2	Ponar	0.5±0.1	1.4±0.1	nd	nd
Grab10	Superior	10/06/10	nd	22.2	Ponar	23.0±0.9	3.4±0.3	nd	nd
CD	Superior	10/05/10	245	8.2	Multi-corer; 0–5 cm	8.8±4.2	5.3±0.2	1.2±0.1	12.4
SteC	Superior	10/07/10	41	10	Multi-corer; 0–5 cm	22.7±2.9	4.3±0.3	nd	11.4
UWM	Superior	10/06/10	171	31	Multi-corer; 0–5 cm	13.2±6.9	12.8±2.3	nd	12.3
WM	Superior	10/06/10	165	56.3	Multi-corer; 0–5 cm	0.9±0.2	5.1±0.4	1.1±0.1	12.5

1) determined in surface water.

2) in the sediment.

3) dissolved oxygen concentration in the water directly above the sediment.

nd: not determined.

### Determination of environmental data

Mineral nitrogen was measured in 1 M KCl extracts. 3–4 g mixed sediment samples were mixed with 1 M KCl in the ratio 1∶10, shaken for 1 h at 200 rpm and centrifuged at 7000 g for 10 min. The supernatant was stored at −20°C until further analysis. Mineral nitrogen (ammonium, nitrite and nitrate) was determined colorimetrically [45]–[48]. Dry weight was determined after drying the sediment samples for 24 h at 110°C.

Water column phytoplankton biomass was assessed using parallel approaches: Chlorophyll *a*, a proxy for biomass, was measured fluorometrically following acetone extraction of seston retained on 0.2 µm polycarbonate filters [49]. Oxygen concentrations were measured during water sampling by a Seabird (Bellevue, WA) model 911 dissolved oxygen sensor.

### Molecular analysis: DNA isolation

DNA was isolated with the PowerSoil DNA isolation kit (MoBIO, Carlsbad, CA, USA) according to the manufacturer's recommendations. DNA concentration was measured spectrophotometrically with a NanoDrop 2000 (Thermo Fisher Scientific, Wilmington, DE). DNA isolation was conducted in triplicate per sample.

### Quantitative PCR (qPCR)

DNA for the qPCR was diluted to concentrations between 1–10 ng/µl. qPCR to determine the abundance of archaeal and bacteria *amoA* genes was performed using AOA and AOB-specific *amoA* primers (Table S1 in File S1; [50], [51]) and the Bioline SensiMix SYBR No-ROX kit according to the manufacturer's recommendations (Bioline, Taunton, MA). Thermocycling was performed using the conditions described in Table S2 and S3 in File S1 using the Illumina Eco Real-Time PCR System (Ilumina, San Diego, CA). The number of gene copies in the samples was calculated using the standard curve method and the specificity of the primers was determined by agarose gel electrophoresis and melting curves (Table S4 in File S1).

### Pyrosequencing of the archaeal and bacteria *amoA* genes

DNA was first amplified with AOA and AOB *amoA* primers (Table S1 in File S1, [50], [51]) under conditions presented in Table S7 and S8 in File S1. The PCR products were diluted 1∶10 and used as template for a second PCR using the barcoded primers (Table S5, Table S6 and Table S9 in File S1). Twelve bar-coded primers were generated, six for archaeal *amoA* and six for bacterial *amoA*, allowing sequencing of the *amoA* genes of six independent samples in 1/16 of a 454 sequencing run. The two-step PCR prevents amplification biases and increases reproducibility [52]. Per DNA isolation, three PCR reactions were conducted and at the end all PCR products (triplicate PCR runs × triplicate DNA isolations) per sample were mixed and used as one sample for pyrosequencing. The samples were purified with AMPureXP (Beckman-Coulter, Inc., Indianapolis, IN, USA). The samples were quantified with PicoGreen assay, diluted, pooled, and purified again with AMPureXP (Beckman-Coulter, Inc., Indianapolis, IN, USA). The concentration in the pooled samples was determined using KAPA qPCR (KAPA Biosystems, Woburn MA, USA). For the first library 0.5 copies per bead and for the second library 2 copies per bead were sequenced on the Roche GS FLX system at the Plant-Microbe Genomics Facility at The Ohio State University (Columbus, Ohio, USA).

### Analysis of the pyrosequencing data (Figure S1)

The data were processed into quality (.qual) and sequence (.fasta) files using GSRmBrowser version 2.5.3. QIIME was used for initial quality filtering of the sequences [53]. The overall sequences files were split based on the barcodes, quality filtered with the average quality score being 25, truncated at 400 bp length and exported as sequence (.fasta) files. The sequences were imported into ARB [54] and translated into protein sequences. The protein sequences were screened to exclude sequences with stop codons and frame shifts. The remaining sequences were exported as nucleotide sequences and further analyzed with QIIME [53]. First the sequences files were merged and the sequences were grouped based on identity into groups with 89% and 98% identity. Since there has been a discussion that the increase in diversity in pyrosequencing libraries could be due to sequencing errors [55], we eliminated all OTU's with just single sequences per sample. The AOA and AOB libraries, respectively, were 100 times rarefied based on the number of sequences in the library with the lowest sequence number (AOA: 538 sequences at 85% and 525 sequences at 98% similarity; AOB: 221 sequences at 85% and 208 sequences at 98% similarity). The rarified libraries were used for the determination of the alpha diversity (number of OTU's, chao1 and Shannon index) and the beta-diversity using two different measures (abundance weighted Jaccard distance and weighted Unifrac analysis), to integrate abundance and phylogenetic information in the beta-diversity analysis. The phylogenetic trees used for the Unifrac analysis were constructed with representative sequences in ARB using the Neighbor-joining method [54].

Representative AOA and AOB sequences based on 98% identity after excluding singletons were aligned to the ARB-AOA file published by Pester et al (2012) [22] and an AOB file and added with the ARB parsimony addition tool [54]. Phylogenetic trees for AOA and AOB were constructed in ARB with the added representative sequences and close related sequences using the Neighbor-joining method.

### Statistical analysis

Correspondence analysis was conducted using CANOCO (http://www.canoco5.com) and statistical analysis (One-Way ANOVA and Pearson correlations) with SPSS (version 19).

The sequences were deposited in the NCBI SRA database under the accession number PRJNA217461 with the individual accession numbers for each library: SRS474329, SRS474331-333, SRS474335-345, SRS474348.

## Results

We analyzed twelve sediment samples from the western basin of Lake Superior (8) and the western basin of Lake Erie (4) (Figure 1, Table 1). Ammonium concentration was by one to two orders of magnitude higher in the sediment samples from Lake Erie than in the samples from Lake Superior, whereas nitrate was by one to two orders of magnitude higher in the Lake Superior samples (Table 1). Chlorophyll *a* in the water column as measure of the trophic state was much higher in the samples from Lake Erie than in the Lake Superior samples (Table 1).

**Figure 2:**
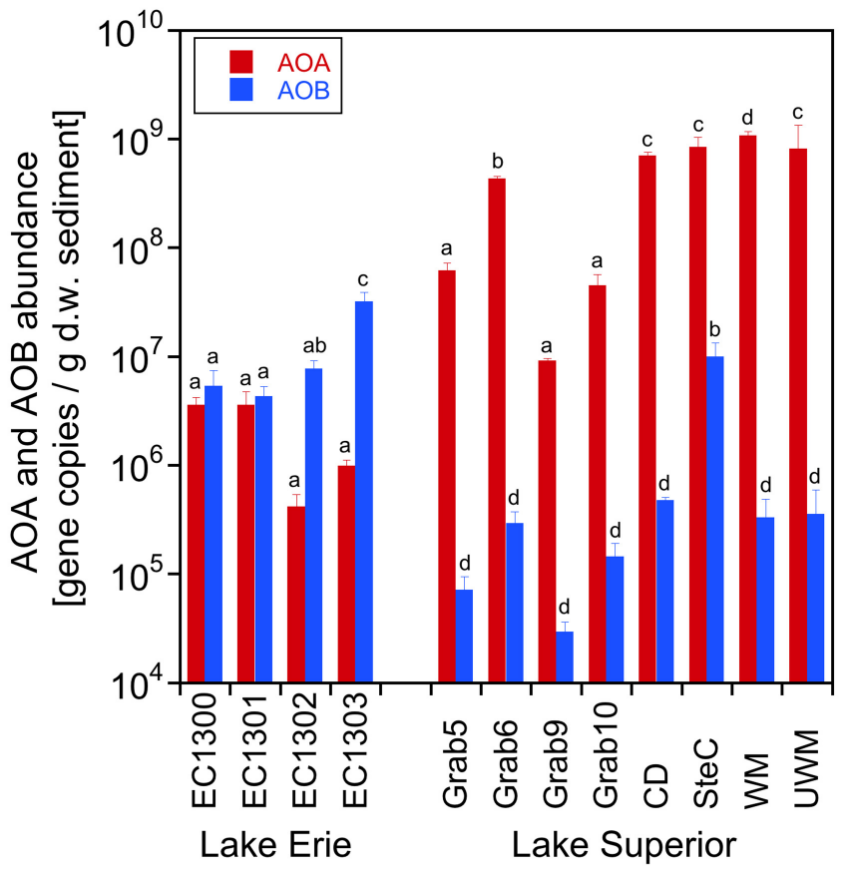
Abundance (*amoA* gene copy number) of AOA and AOB in the sediment of Lake Erie and Superior (mean ± SD, n = 3; different letters above the columns indicate significant differences between samples determined by one-way ANOVA followed by Tukey test; p<0.05).

The abundance of AOA and AOB in the sediment was determined using AOA and AOB specific *amoA* primers (Figure 2). In the samples EC1300 and EC1301 from Lake Erie's Maumee Bay, AOA and AOB abundances were in the same order of magnitude. AOB were by one order of magnitude more abundant than AOA in the samples EC1302 and EC1303 from Sandusky Bay showing a dominance of the AOB in all samples from Lake Erie. By contrast, AOA were 2–4 orders of magnitude more abundant than AOB in Lake Superior. In summary, sediment samples from embayments of Lake Erie were dominated by AOB, whereas Lake Superior samples were dominated by AOA.

The archaeal and bacterial *amoA* genes were sequenced using a pyrosequencing approach in two separate runs. AOB *amoA* genes were only sequenced from the four samples from Lake Erie, because the AOB abundance in Lake Superior was very low. Run 1 resulted in 11847 sequences after sequencing and 6668 sequences after quality control with QIIME and ARB whereas run 2 resulted in 20892 and 13563 sequences, respectively (Table S10 in File S1).

**Figure 3:**
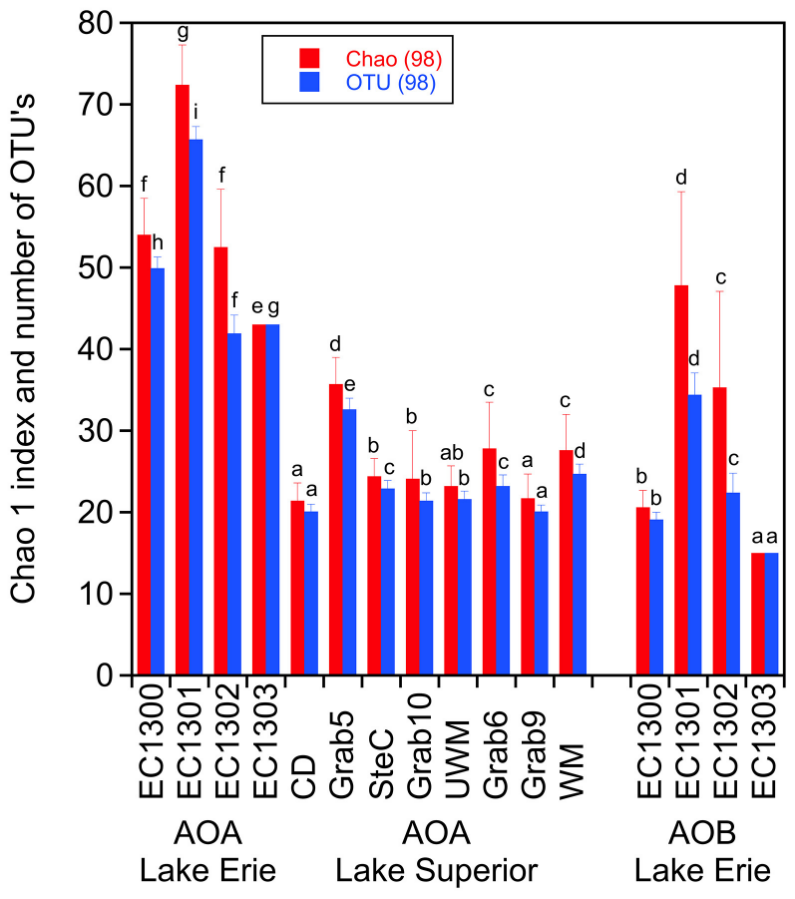
Alpha-diversity at 98% identity and singletons removed of the AOA and AOB *amoA* sequence libraries. (mean ± SD, n = 100 rarefactions; different letters in figure A above the columns indicate significant differences between samples determined by one-way ANOVA followed by Tukey test; p<0.05; data for AOA and AOB were tested separately).

The number of OTU's, the Chao1 index (richness) and the Shannon index (evenness) were calculated at cutoff values of 85% and 98% similarity using the software package QIIME [53] (Figure 3; Table S11, Table S12 and Table S13 in File S1). The number of OTU's and the Chao1 index for the AOA were at both cutoff values higher for the samples from Lake Erie (OTU's cutoff 85%: 9–12 and cutoff 98%: >40) than for the samples from Lake Superior (OTU's cutoff 89%: 2–7 and cutoff 98%: around 20) indicating a higher AOA diversity in Lake Erie than in Lake Superior (Figure 3; Table S11 and Table S12 in File S1). The evenness of the AOA in the samples from Lake Erie was higher than in Lake Superior, as shown by the higher Shannon index in the samples from Lake Erie compared to Lake Superior (Table S13 in File S1). The number of OTU's for AOB in Lake Erie ranged from 7–12 (89% similarity) and 15–40 (98% similarity). The chao1 indices for the AOB were similar or a little higher than the OTU numbers for the AOB in Lake Erie (Fig. 3; Table S11 and Table S12 in File S1).

AOA and AOB abundances were correlated using Pearson correlation to environmental factors and alpha-diversity data (Table 2). The AOA abundance was positively correlated with nitrate concentration (p<0.05) while the AOB abundance was positively correlated with ammonium concentration in the sediment (p<0.05). Chlorophyll *a* concentration in the water column as an indicator of the trophic state of the lakes showed positive correlation with the AOB abundance (p<0.05) and negative correlation with the AOA abundance (p<0.05) indicating that AOA are more prevalent under low nutrient oligotrophic conditions and AOB under nutrient rich meso- to eutrophic conditions. Of note, the AOA abundance showed negative correlation with alpha diversity; high AOA abundances in the Lake Superior samples coincided with low species diversity and low AOA abundances in the Lake Erie samples with high species diversity (Figure 2; Figure 3; Table 2; Table S11 and Table S12 in File S1).

**Table 2:** Pearson correlations of AOA and AOB abundances with environmental and alpha diversity data.

	AOA abundance	AOB abundance	n =
Ammonium (sediment)	−0.344	0.734*	12
Nitrate (sediment)	0.906*	−0.445	12
Chlorophyll A (watercolumn)	−0.930*	0.912*	6
Chao1 index	−0.744*	−0.756	12/4
Number of OTU's	−0.741*	−0.790	12/4
Shannon index	−0.591*	−0.372	12/4

* p<0.05. (All data log-transformed.)

**Figure 4:**
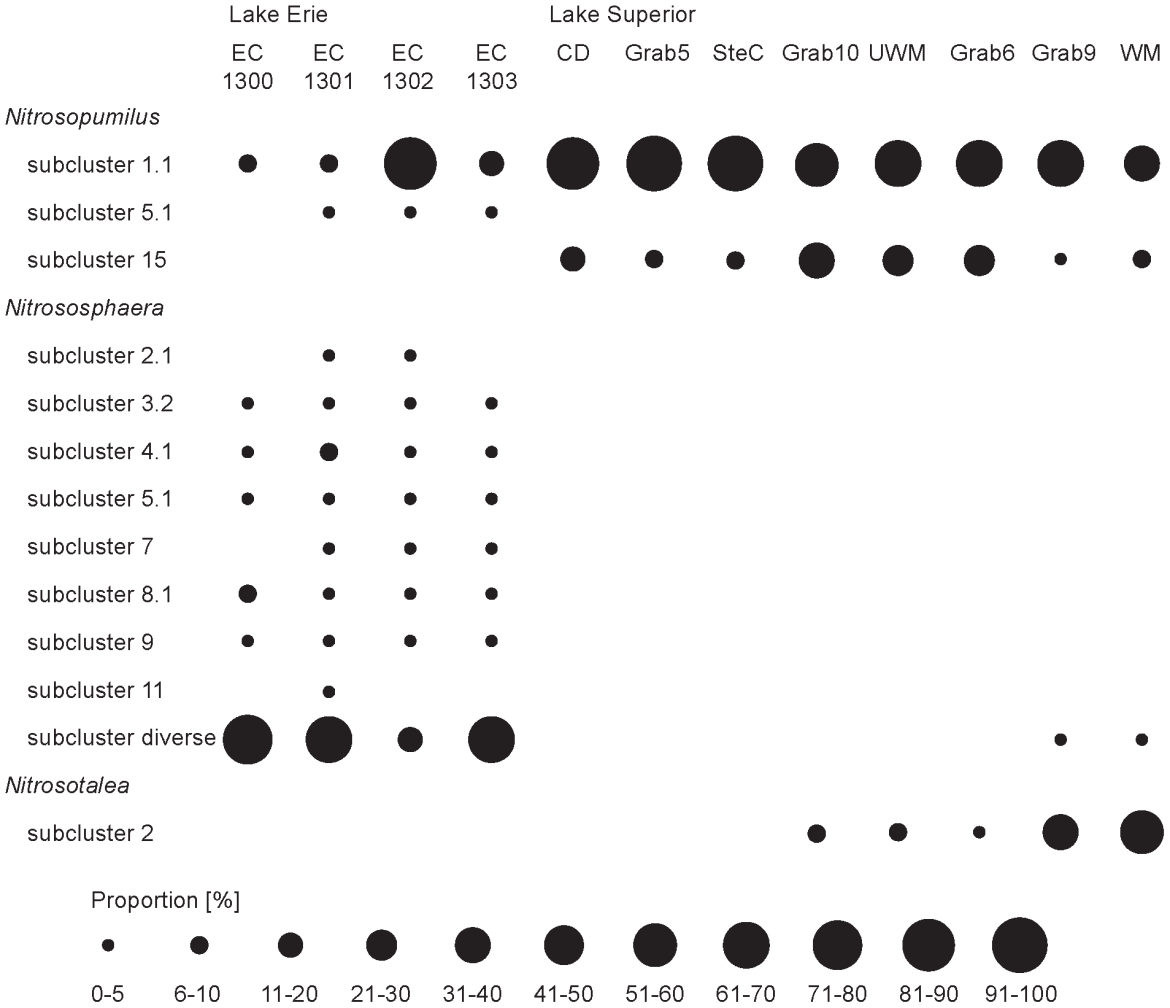
Relative frequency [%] of the AOA in the different phylogenetic groups. Representative sequences were picked based on 98% identity, aligned in ARB to the AOA tree published by Pester et al. (2012) and assigned to different phylogenetic groups. Cultivated members of the AOA can be found in *Nitrosopumilus* cluster 1.1: *Nitrosopumilus maritimus*, *Candidatus* Nitrosoarchaeum limnia, *Candidatus* Nitrosoarchaeum korensis MY1 and Enrichment AOA-AC2; *Nitrosopumilus* cluster 5: Enrichments AOA-AC1, AOA-AC5 and AOA-DW. Most cultivated strains are integrated in Figure S3.

Phylogenetic affiliation of AOA and AOB was determined by aligning representative sequences (98% similarity; 400 bp length) in ARB. AOA were classified using the classification published by Pester et al., 2012 (Figure 4; Figure S2). AOA sequences in Lake Superior and Lake Erie exhibited very different community compositions (Figure 4). The AOA communities in Lake Erie were dominated by a wide variety of *Nitrososphaera*-like sequences, except sample EC1302 from the mouth of Sandusky Bay that contained a high number of *Nitrosopumilus*-like sequences. All other Lake Erie samples contained only low numbers of *Nitrosopumilus* sequences. Sequences from the group *Nitrosopumilus* subcluster 1.1 were found in all samples from Lake Superior in high quantities (>50% of total sequences). In addition members of *Nitrosopumilus* subcluster 15 were detected in quantities higher than 10% in samples from Grab6, Grab10, and UWM and *Nitrosotalea* cluster 2 in samples from Grab9 and WM. Only in the samples Grab9 and WM were low numbers of *Nitrososphaera* sequences detected. A detailed phylogenetic tree showed that only two sequences in the *Nitrososphaera* cluster were more than 5% abundant (Sequence 59 and 88 in samples EC1300, EC1301, EC1303). All other sequences were detected in lower abundances (<5% of the total sequences). The sequences from Lake Erie clustering in *Nitrosopumilus* cluster 1.1 were different from the sequences found in Lake Superior (Figure S2). Sequences from the *Nitrosopumilus* cluster 5.1/5.2, a *Nitrosopumilus* cluster dominated by freshwater and ground water sequences, were only detected in Lake Erie (Figure 4; Figure S2).

**Figure 5:**
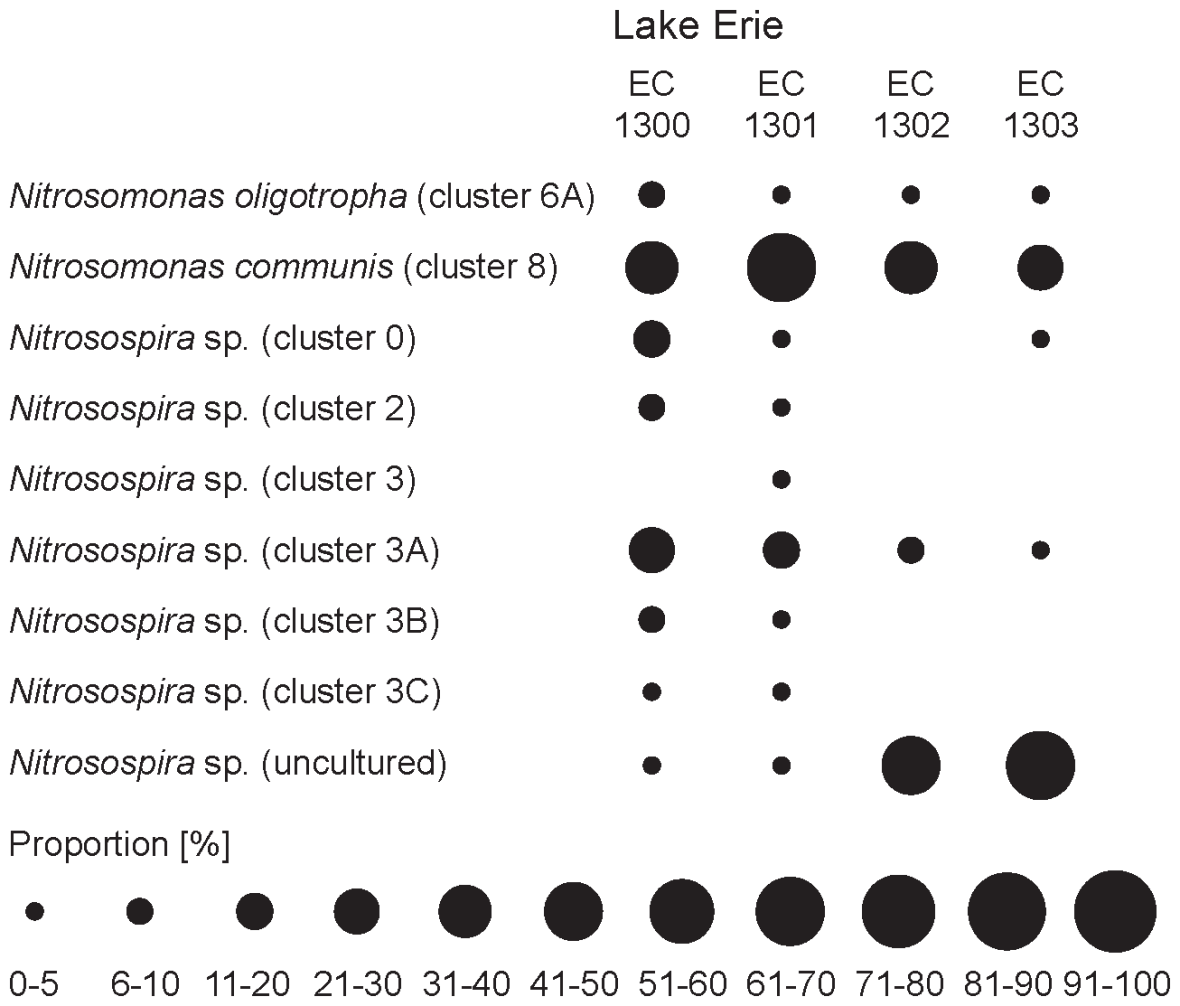
Relative frequency [%] of the AOB in the different phylogenetic groups. Representative sequences were picked based on 98% identity, aligned in ARB and assigned to phylogenetic groups.

The AOB community in Lake Erie was dominated by members of the *Nitrosomonas communis* cluster (*Nitrosomonas* cluster 8) and the *Nitrosospira* cluster (*Nitrosospira* cluster 0; 3A and uncultured) (Figure 5; Figure S3). Only low numbers (up to 10% of the total sequences) of sequences were found in the typical freshwater cluster *Nitrosomonas* cluster 6a. The Maumee Bay samples EC1300 and EC1301 contained *Nitrosomonas communis* and *Nitrosospira* cluster 3A sequences as well as low numbers from other *Nitrosospira* clusters. The AOB communities in the Sandusky Bay samples from EC1302 and EC1303 were less diverse, as only 2-3 different *Nitrosospira* clusters could be detected.

**Figure 6:**
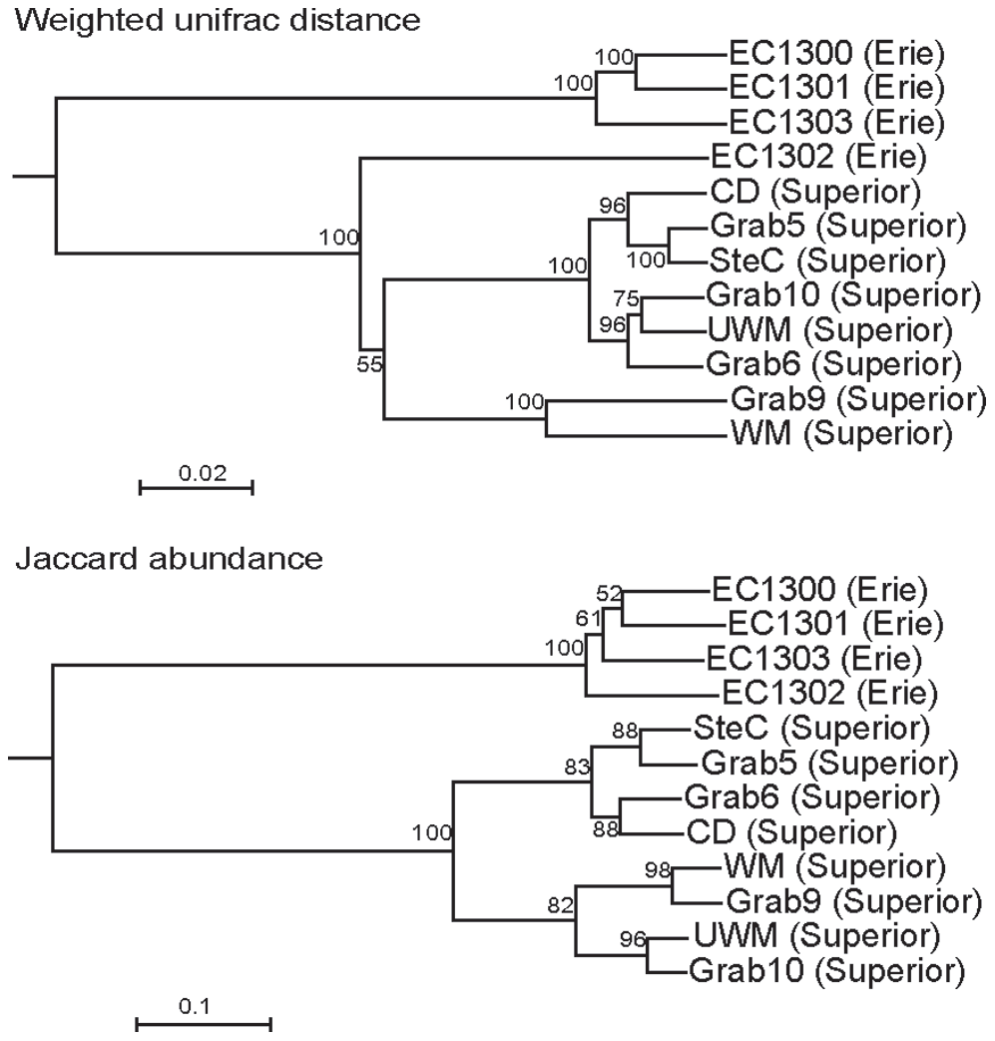
UPGMA clustering of Weighted Unifrac distances (A) and Jaccard abundances (B) of AOA in the sediment of Lake Erie and Superior at 98% similarity. Numbers at the nodes represent statistical analysis of 100 rarefactions.

The community composition of AOA was compared using Weighted Unifrac distance and Jaccard abundance [53], [56]. Weighted Unifrac distance analysis is based on phylogenetic relationship and abundance, while Jaccard abundance is based only on abundance. Overall both analyses showed similar relationships between the different communities (Figure 6). Samples from Lake Superior and samples from Lake Erie, respectively, clustered together with the exception of the sample EC1302 from Lake Erie. This sample clustered together with the Lake Erie samples when analyzed with Jaccard abundance but with Lake Superior samples when analyzed with Weighted Unifrac distance. Sample EC1302 exhibited a high number of sequences belonging to the *Nitrosopumilus* subcluster 1.1 while the other Lake Erie samples contained low numbers and the Lake Superior samples high numbers of sequences from that cluster (Figure 4; ). The AOB communities of EC1300 and EC1301 as well as EC1302 and EC1303 clustered together based on Weighted Unifrac analysis (Figure S4).

**Figure 7:**
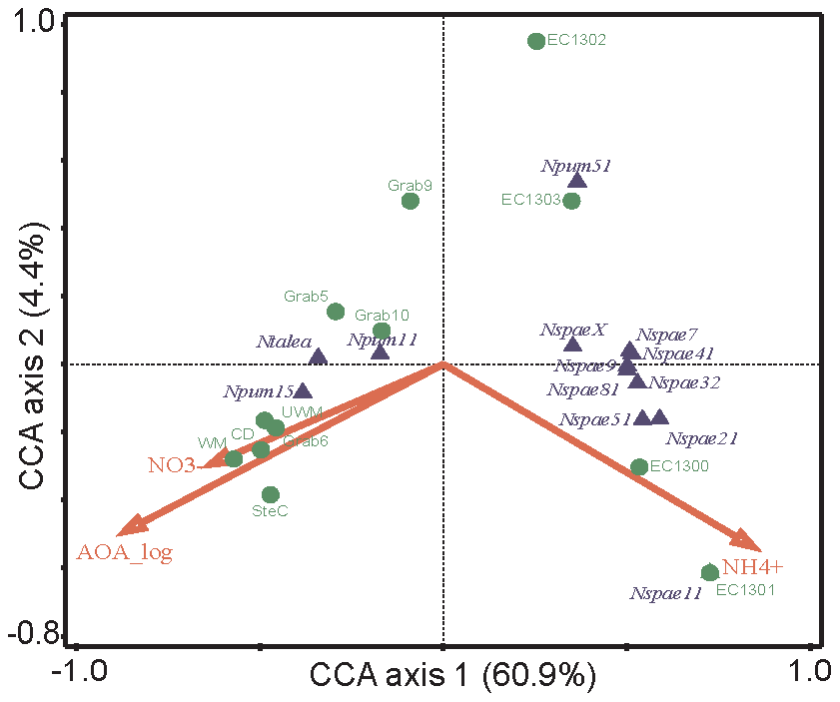
Canonical correspondence analysis (CCA) triplot (arrows: environmental variables; circles: samples; triangles: species) for quantitative data as presented in Fig. 3 of the AOA *amoA* sequence libraries in Lake Erie and Superior (Eigenvalues: Axis 1: 0.5865, Axis 2: 0.0421, Axis 3: 0.0073). Explanatory value of the environmental factors was determined using forward selection: AOA_log explained 47%; ammonium concentration 18.3% and nitrate concentration 0.8% of the variation. (Abbreviations: Npum11: *Nitrosopumilus* subcluster 1.1; Npum51: *Nitrosopumilus* subcluster 5.1; Npum15: *Nitrosopumilus* subcluster 15; Nspae21: *Nitrososphaera* subcluster 2.1; Nspae32: *Nitrososphaera* subcluster 3.2; Nspae41: *Nitrososphaera* subcluster 4.1; Nspae51: *Nitrososphaera* subcluster 5.1; Nspae7: *Nitrososphaera* subcluster 7; Nspae81: *Nitrososphaera* subcluster 8.1; Nspae9: *Nitrososphaera* subcluster 9; Nspae11: *Nitrososphaera* subcluster 11; NspaeX: *Nitrososphaera* subcluster diverse; Ntalea: *Nitrosotalea* subcluster 2)

Canonical correspondence analysis (CCA) was used to determine relationships between species, communities and the environmental factors (Figure 7; Figure S5). The AOA communities from Lake Superior were positive related with AOA abundance and nitrate concentration and from Lake Erie to ammonium concentration (Figure 7). The AOA abundance explained 47% and the ammonium 18.3% of the variation. Ammonium concentration and AOB abundance were the most important factors explaining the AOB community composition (Figure S5).

## Discussion

### Niche differentiation between AOA and AOB in freshwater environments

AOA were more abundant in the sediments of oligotrophic Lake Superior than AOB whereas the situation in two meso-eutrophic embayments of Lake Erie was vice versa (Figure 2). The AOA abundance was negatively, and the AOB abundance positively correlated with chlorophyll *a* concentration – an indirect measure of the trophic state of the lakes (Table 2). Our results are consistent with recent reports showing dominance by AOA over AOB in the water column of two oligotrophic lakes, Lake Superior [43] and Lake Lucerne [37]. Likewise, AOA are the dominant ammonia oxidizers in the open ocean where nutrients are scarce [57]-[59]. By contrast, the ratio of AOB to AOA increases with increasing ammonium concentrations in freshwater streams [33] and soils [3]. These results confirm the trend that AOA are found in more nutrient poor and AOB in nutrient rich environments.

The ammonium concentration was one of the major factors regulating abundance and distribution of the AOA (Table 2; Figure 7). In freshwater aquarium biofilters and in a wastewater treatment plant, the AOA abundance was negatively related to the ammonium concentration [60], [61]. AOA enrichment cultures and the pure culture *Nitrosopumilus maritimus* have much lower K_m_ values for ammonium/ammonia than AOB [10]–[12]. Overall these results show that the majority of AOA can be found in and is very likely adapted to environments with low ammonium concentrations and availability.

Based on the abundances of and ratios between AOA and AOB in the oligotrophic Lake Superior and meso/eutrophic Lake Erie and under the assumption that AOA and AOB use primarily ammonia oxidation for energy generation it is likely that AOA were mainly responsible for ammonia oxidation in Lake Superior and AOB in Lake Erie. Nitrification in the water column in Lake Superior has been attributed to the activity of AOA, which were found to be the dominant ammonia oxidizers in that environment [43]. This observation and the high abundance of AOA in the sediments of Lake Superior support our assumption that AOA are very likely the main ammonia oxidizers in the sediments of Lake Superior.

### Niche differentiation between different groups of AOA

The results showed not only a niche differentiation between AOA and AOB, but also between different groups of AOA (Figure 4). In 3 out of 4 Lake Erie samples members of the *Nitrososphaera* soil/sediment cluster and *Nitrosopumilus* cluster 5 were the dominant AOA while the Lake Superior samples were dominated by members of *Nitrosopumilus* cluster 1 and 15 and the *Nitrosotalea* cluster (Figure 4). Also these differences could be due to the trophic states of the two lakes. Sequences from the *Nitrosopumilus* cluster 1.1 cluster together with sequences from the Qiantang River in China [34], the rhizosphere of the freshwater macrophyte *Littorella uniflora* [31], oligotrophic freshwater lakes [14], [62] and a drinking water distribution system [63]. The freshwater enrichment cultures AOA-AC2 and *Candidatus* Nitrosoarchaeum limnia belong to the same AOA subcluster[11], [15], [64].

High proportions of AOA *amoA* sequences from Lake Superior were detected in the cluster *Nitrosopumilus* cluster 15. Interestingly this cluster has not been dominated by freshwater strains. Most sequences were found in estuarine sediments such as Douro River estuary (Portugal) [65], Plum Island Sound [8], the Elkhorn Slough [50] and also in high altitude lakes of the Tibetan Plateau [66].

Finally many sequences from Lake Superior were detected in the *Nitrosotalea* cluster. Sequences from both *Nitrosotalea* clusters have been found frequently in oligotrophic freshwater [14], [29], [31], [32], groundwater [63], [67] and acidic soils [6], [68]. The only cultivated member of the *Nitrosotalea* cluster is *Candidatus* Nitrosotalea devanaterra, an obligate acidiphilic AOA [69]. However, the pH in Lake Superior waters average 7.2, indicating that acidophilic conditions were not the cause for the high abundance of members of the *Nitrosotalea* cluster in these sediments.

Overall many sequences in the *Nitrosopumilus* cluster 1.1 and the *Nitrosotalea* cluster have been detected in rather nutrient poor systems, an observation that is in accordance with our observations that *Nitrosopumilus* cluster 1.1 and the *Nitrosotalea* cluster are highly abundant in the sediment samples of the oligotrophic Lake Superior.

In Lake Erie a few sequences from *Nitrosopumilus* cluster 5 were detected. *Nitrosopumilus* cluster 5 was found in other freshwater environments such as Lake Taihu [70], groundwater [63], [67], freshwater sediments [31], [34], roots of macrophytes [32], aquarium filters [60], a wastewater treatment plant [28] and enriched from the sediments of two meso/eutrophic lakes in Ohio [15] and hot springs [71]. Overall, sequences clustering in this group were detected in meso-to-eutrophic environments rather than in oligotrophic environments. This observation indicates that freshwater *Nitrosopumilus* cluster 5 strains could be adapted to different trophic states in their environment than the AOA strains detected in Lake Superior.

In addition many AOA sequences in Lake Erie belonging to the *Nitrososphaera* cluster I.1b were related to sequences from soils samples. The sampling sites in Lake Erie were close to the mouth of two rivers with agricultural watersheds. The presence of these sequences could have different reasons: (1) *Nitrososphaera*-like AOA in the eutrophic sediments could coexist with the AOB due to spatial separation; (2) some *Nitrososphaera*-like AOA sequences/strains could have originated from agricultural runoff rather than exist as members of the active ammonia-oxidizing community in those sediments; or (3) AOA could have additional metabolic capabilities providing them with an advantage over AOB (i.e. mixotrophy or non-autotrophic growth) [27], [28]. Mixotrophic growth was observed in AOA from both large groups (*Nitrosopumilus* cluster I.1.a and *Nitrososphaera* cluster I.1.b) [72], [73] and AOA belonging to the *Nitrososphaera* cluster I.1.b found in a refinery wastewater sample did express *amoA* but did not oxidize ammonia [28].

An additional important difference between the samples from the two lakes is that the overall AOA abundance in Lake Superior is by several orders of magnitude higher than in Lake Erie while the overall diversity of the AOA is lower (Figure 2; Figure 3; Table 2; Table S11 and Table S12 in File S1). Similar observations have been made in other oligotrophic freshwater environments [14], [32], [62] and peatland soil [74]. In all those environments were AOA much more abundant than AOB, while the AOA diversity was rather low.

### AOB phylogeny

In Lake Erie the AOB are as or more abundant than the AOA (Figure 2). Members of the *Nitrosomonas communis* cluster were found in high abundance in all samples from Lake Erie whereas *Nitrosomonas oligotropha*-like AOB were only present in low abundances (Figure 5; Figure S3). *N. communis* is found typically in eutrophic-, and *N. oligotropha* in oligotrophic freshwater environments [20], [75]-[77] indicating that the dominant *Nitrosomonas* strains reflect the trophic state of the Lake Erie.

### Pyrosequencing of AOA and AOB *amoA* genes

Molecular surveys of AOA and AOB use 16S rRNA or *amoA* genes as marker genes [50], [51], [78]. Up to now most studies used a cloning-sequencing approach rather than a pyrosequencing approach to access the *amoA* diversity. Only a few studies have already used pyrosequencing of the *amoA* gene to describe AOA and AOB diversity [22], [68], [79], [80]. One of the major problems with pyrosequencing is the quality control of the sequences to ensure that the diversity is not overestimated based on sequencing errors [55]. The study demonstrated inflated 16SrDNA diversity due to a non-stringent quality control of the sequences. However, the use of functional genes has the advantage that the genes can be analyzed on the nucleotide- and the protein-level to eliminate sequences with sequencing errors more effectively [81]. During the ARB step (conversion into proteins) in the quality control around 30–40% of the sequences were removed due to the presence of stop codons and frame shift in the sequences (Table S10 in File S1). The presence of these sequences in the libraries for analysis increased the overall diversity in the samples artificially (results not shown) demonstrating the need for rigorous quality control of pyrosequencing data using functional genes. One downside of eliminating the sequences with frame shifts and stop codons could be to eliminate not just sequences with sequence errors, but also sequences of non-functional gene copies. However, this is rather unlikely to be a large problem, because most of the sequences that were removed had the stop codons and frame shifts at the end of the sequence reads or were single sequences with mistakes resulting in the conclusion that these errors are very likely due to sequencing mistakes.

### Conclusions and Outlook

AOA dominated the sediments in Lake Superior and AOB in Lake Erie. In addition differences in the community composition of the AOA were observed between Lake Superior and Lake Erie. Due to the environmental conditions and the abundances of AOA and AOB in both lakes, it can be concluded that trophic state of the environment and ammonium availability play a key role in the niche differentiation between AOA and AOB as well as between the different groups of AOA in Lake Erie and Superior. Based on these observations future experiments should include the enrichment of new freshwater AOA-strains, investigation of the niche differentiation between AOA and AOB, as well as between different groups of AOA, and investigation of the basic physiology in connection with the main environment from which the strains were obtained to get better insights into the physiological capacities of AOA and AOB in freshwater systems.

## Supporting Information

Figure S1 **Overview over sequence analysis.**
(TIFF)

Figure S2 **Neighbor-joining tree of the AOA** ***amoA*** **nucleotide sequences.**
(TIF)

Figure S3 **Neighbor-joining tree of the AOB** ***amoA*** **nucleotide sequences.**
(TIF)

Figure S4 **UPGMA clustering of weighted unifrac distance of the AOB** ***amoA*** **sequences in Lake Erie.**
(TIF)

Figure S5 **Canonical correspondence analysis (CCA) of AOB** ***amoA*** **sequences in Lake Erie.**
(TIF)

File S1 **Combined file containing tables S1 – S13.**
(DOCX)
