# Supplementary figures and images for: Abundance and Diversity of Ammonia-Oxidizing Archaea and Bacteria in Sediments of Trophic End Members of the Laurentian Great Lakes, Erie and Superior
Source: PLoS One. 2014 May 12;9(5):e97068. doi: 10.1371/journal.pone.0097068 (PMC4018257; doi:10.1371/journal.pone.0097068)

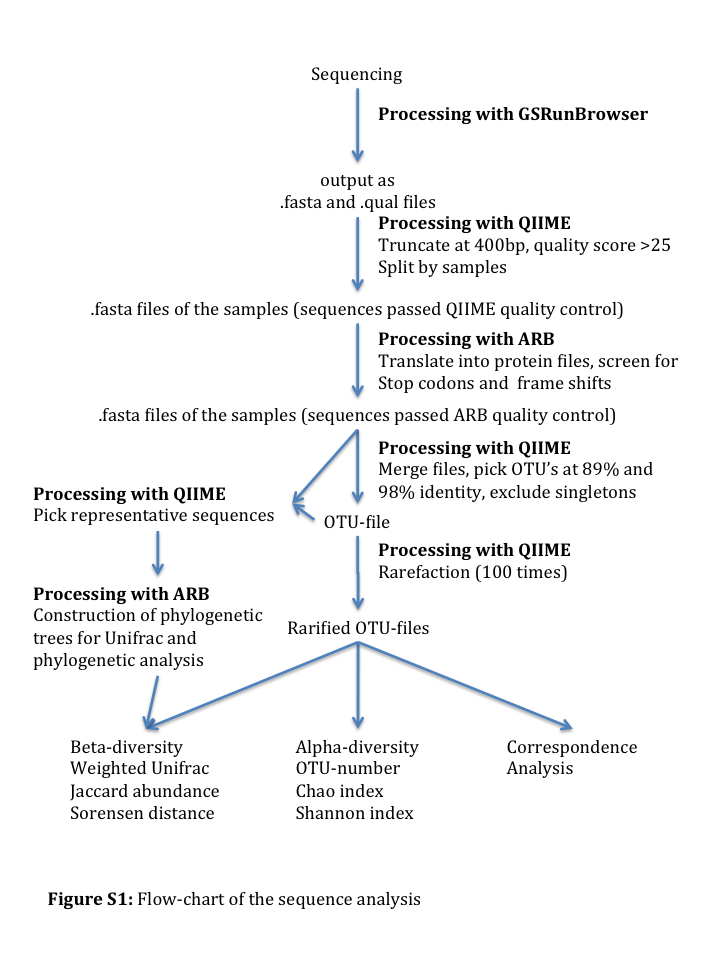

Supplement: Figure S1 — Overview over sequence analysis. (TIFF) [file pone.0097068.s001.tif]

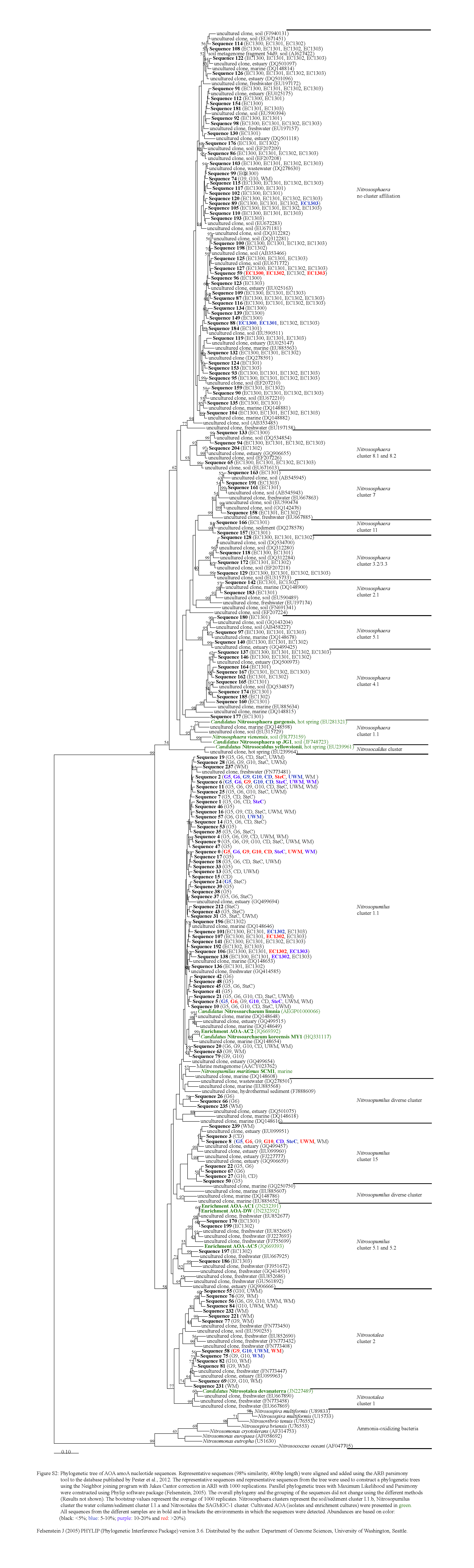

Supplement: Figure S2 — Neighbor-joining tree of the AOA amoA nucleotide sequences. (TIF) [file pone.0097068.s002.tif]

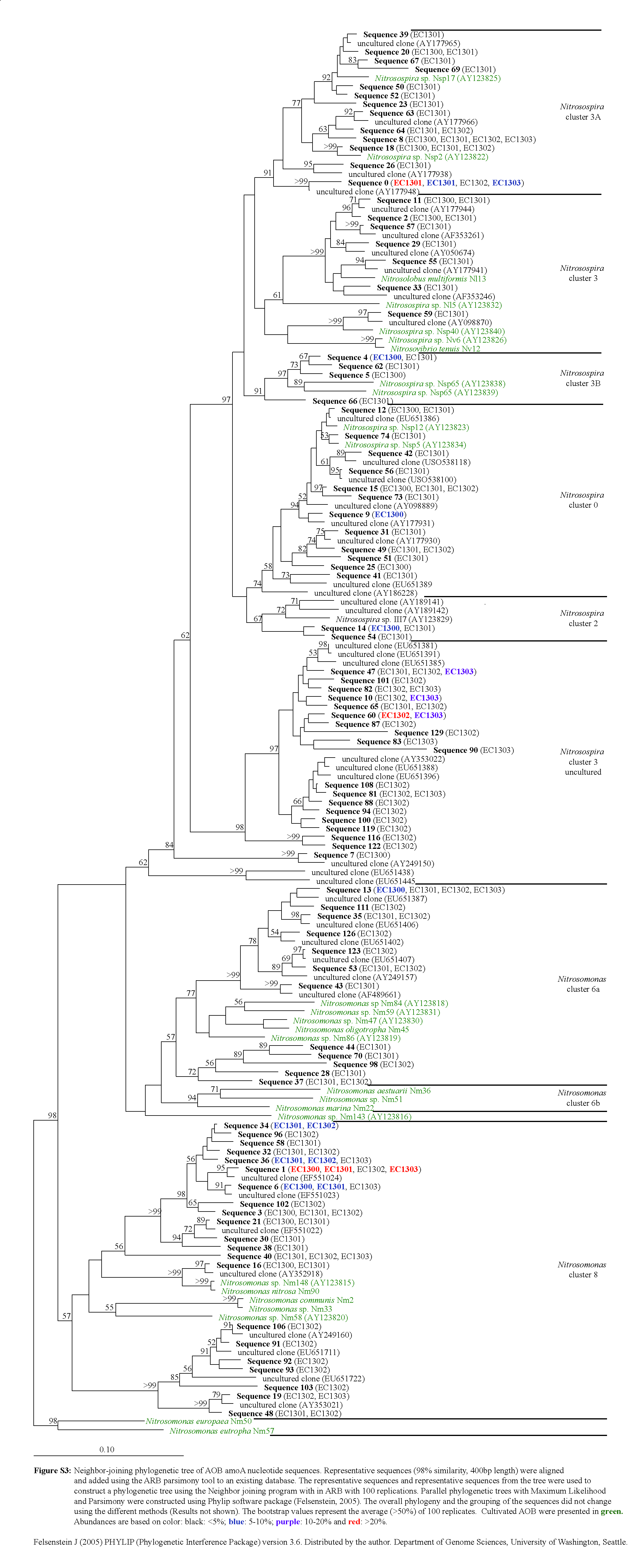

Supplement: Figure S3 — Neighbor-joining tree of the AOB amoA nucleotide sequences. (TIF) [file pone.0097068.s003.tif]

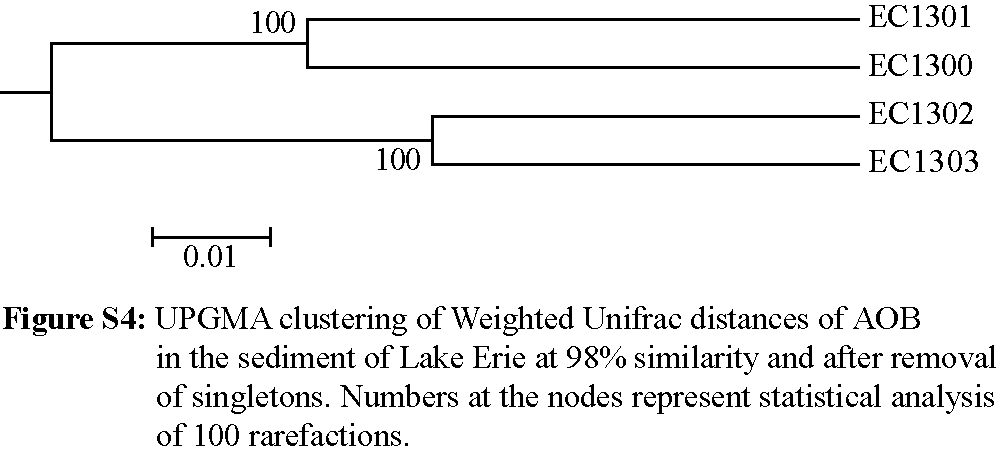

Supplement: Figure S4 — UPGMA clustering of weighted unifrac distance of the AOB amoA sequences in Lake Erie. (TIF) [file pone.0097068.s004.tif]

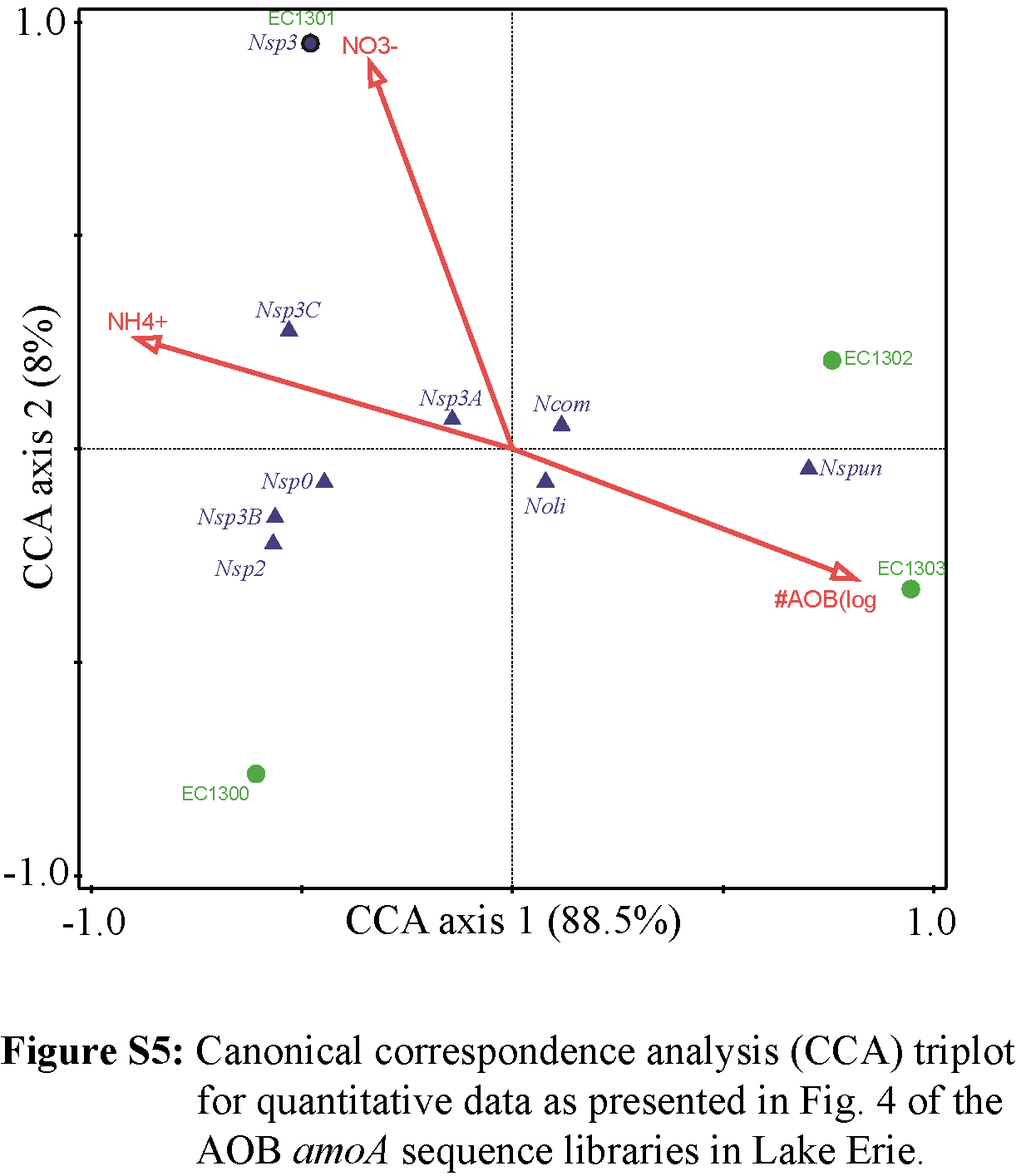

Supplement: Figure S5 — Canonical correspondence analysis (CCA) of AOB amoA sequences in Lake Erie. (TIF) [file pone.0097068.s005.tif]
