# Supplementary material for: Abundance and Diversity of Ammonia-Oxidizing Archaea and Bacteria in Sediments of Trophic End Members of the Laurentian Great Lakes, Erie and Superior
Source: PLoS One. 2014 May 12;9(5):e97068. doi: 10.1371/journal.pone.0097068 (PMC4018257; doi:10.1371/journal.pone.0097068)
Supplement: File S1 — Combined file containing tables S1 – S13. (DOCX) [file pone.0097068.s006.docx]

**TABLE S1:** Primers used in this study.

|  | Primer | Amplicon  length [bp] |
| --- | --- | --- |
| AOA-*amoA*  (Francis et al., 2005) | Arch *amoA* F: 5’-STAATGGTCTGGCTTAGACG-3’  Arch *amoA* R: 5’-GCGGCCATCCATCTGTATGT-3’ | 635 |
| AOB-*amoA*  (Rotthauwe et al., 1997) | *amoA*-1F: 5’-GGGGTTTCTACTGGTGGT-3’  *amoA*-2R KS: 5’-CCCCTCKGSAAAGCCTTCTTC-3’ | 491 |

**TABLE S2:** PCR conditions for qPCR (quantitative PCR); primers from Table S1.

|  | Temperature [°C] | Time (AOA) [s] | Time (AOB) [s] |
| --- | --- | --- | --- |
| Denaturation (initial) | 95 | 600 | 600 |
| Denaturation | 95 | 30 | 30 |
| Annealing | 53 (AOA), 55.4 (AOB) | 45 | 60 |
| Extension | 72 | 60 | 60 |
| Cycles |  | 45 | 45 |
| Melting curve | 95 |  |  |
|  | 55 |  |  |

**TABLE S3:** PCR mix for qPCR; primers from Table S1.

|  | qPCR |
| --- | --- |
| qPCR master mix (2x) | 5 µl |
| Water | 2.6 µl |
| Primer (10 µM) | 0.2 µl |
| DNA template | 2 µl |
|  | Isolated DNA  (1:10 – 1:100 diluted) |

**TABLE S4:** Validation of AOA and AOB *amoA* qPCR

|  | AOA *amoA* qPCR | AOB *amoA* qPCR |
| --- | --- | --- |
| Calibration [copies/µl] | 6×10^2^ - 6×10^6^ | 2.77×10^2^ – 2.77×10^6^ |
| R^2^ | 0.99 | 0.99 |
| Efficiency [%] | 93.3 - 95.9 | 92.7 - 93.4 |

**TABLE S5:** AOA-*amoA* primers (blue) (Francis et al., 2005) with bar codes (red) and linker to the 454 sequencing plate (black)

| Primer |  |
| --- | --- |
| AOA-F1 | CCA TCT CAT CCC TGC GTG TCT CCG ACT CAG ACA CGA CGA CTS TAA TGG TCT GGC TTA GAC G |
| AOA-F2 | CCA TCT CAT CCC TGC GTG TCT CCG ACT CAG ACA CGT AGT ATS TAA TGG TCT GGC TTA GAC G |
| AOA-F3 | CCA TCT CAT CCC TGC GTG TCT CCG ACT CAG ACA CTA CTC GTS TAA TGG TCT GGC TTA GAC G |
| AOA-F4 | CCA TCT CAT CCC TGC GTG TCT CCG ACT CAG ACG ACA CGT ATS TAA TGG TCT GGC TTA GAC G |
| AOA-F5 | CCA TCT CAT CCC TGC GTG TCT CCG ACT CAG ACG AGT AGA CTS TAA TGG TCT GGC TTA GAC G |
| AOA-F6 | CCA TCT CAT CCC TGC GTG TCT CCG ACT CAG ACG CGT CTA GTS TAA TGG TCT GGC TTA GAC G |
| AOA-R | CCT ATC CCC TGT GTG CCT TGG CAG TCT CAG GCG GCC ATC CAT CTG TAT GT |

**TABLE S6:** AOB-*amoA* primers (blue) (Rotthauwe et al., 1997) with bar codes (red) and linker to the 454 sequencing plate (black).

| Primer |  |
| --- | --- |
| AOB-F7 | CCA TCT CAT CCC TGC GTG TCT CCG ACT CAG ACG TAC ACA CTG GGG TTT CTA CTG GTG GT |
| AOB-F8 | CCA TCT CAT CCC TGC GTG TCT CCG ACT CAG ACG TAC TGT GTG GGG TTT CTA CTG GTG GT |
| AOB-F9 | CCA TCT CAT CCC TGC GTG TCT CCG ACT CAG ACG TAG ATC GTG GGG TTT CTA CTG GTG GT |
| AOB-F10 | CCA TCT CAT CCC TGC GTG TCT CCG ACT CAG ACT ACG TCT CTG GGG TTT CTA CTG GTG GT |
| AOB-F11 | CCA TCT CAT CCC TGC GTG TCT CCG ACT CAG ACT ATA CGA GTG GGG TTT CTA CTG GTG GT |
| AOB-F12 | CCA TCT CAT CCC TGC GTG TCT CCG ACT CAG ACT CGC GTC GTG GGG TTT CTA CTG GTG GT |
| AOB-R | CCT ATC CCC TGT GTG CCT TGG CAG TCT CAG CCC CTC KGS AAA GCC TTC TTC |

**TABLE S7:** PCR mix for regular PCR and PCR with barcoded primers; primers from Table S1, S5, and S6.

|  | Regular PCR | PCR with barcoded primers |
| --- | --- | --- |
| Master mix (2x) | 12.5 µl | 12.5 µl |
| Water | 10.5 µl | 11 µl |
| Primer (10 µM) | 0.5 µl | 0.25 µl |
| DNA template | 1 µl | 1 µl |
|  | Isolated DNA | PCR product from regular PCR; 1:10 diluted in water. |

**TABLE S8**: PCR conditions for regular PCR for AOA and AOB; primers from Table S1.

|  | Temperature [°C] | Time (AOA) [s] | Time (AOB) [s] |
| --- | --- | --- | --- |
| Denaturation (initial) | 95 | 300 | 300 |
| Denaturation | 95 | 45 | 30 |
| Annealing | 53 (AOA), 55.4 (AOB) | 60 | 90 |
| Extension | 72 | 60 | 90 |
| Final extension | 72 | 900 | 600 |
| Cycles |  | 35 | 35 |

**TABLE S9**: PCR conditions for *amoA* PCR for AOA and AOB with bar coded primers; primers from Table S5 and Table S6.

|  | Temperature [°C] | Time (AOA) [s] | Time (AOB) [s] |
| --- | --- | --- | --- |
| Denaturation (initial) | 95 | 300 | 300 |
| Denaturation | 95 | 45 | 30 |
| Annealing | 53 (AOA), 55.4 (AOB) | 90 | 90 |
| Extension | 72 | 60 | 90 |
| Final extension | 72 | 900 | 600 |
| Cycles |  | 10 | 10 |

**TABLE S10:** Number of sequences per pyrosequencing run before and after quality filtering

|  | Run1 | Run2 |
| --- | --- | --- |
| After GS browser | 11847 | 20892 |
| After QIIME QC | 10916 | 19271 |
| AOA | 9152 | 10932 |
| AOB | 1764 | 8339 |
| After ARB QC | 6668 (61.1%) | 13563 (70.4%) |
| AOA | 6085 (66.5%) | 7619 (69.7%) |
| AOB | 583 (33.0%) | 5944 (71.2%) |

**TABLE S11:** Chao1 index of AOA and AOB communities in the sediments of Lake Erie and Lake Superior. The OTU’s were picked per community using different identity cutoffs (85% and 98%), the communities were rarefiyed and alpha diversity was calculated (mean±SD, n=100)

| Identity |  | 85% | | 98% | |
| --- | --- | --- | --- | --- | --- |
| Singletons |  | + | - | + | - |
| AOA (Erie) | EC1300 | 8.9±0.5 | 8.9±0.5 | 80.3±14.8 | 54.0±4.5 |
|  | EC1301 | 12.8±1.6 | 12.0±0.9 | 94.8±13.1 | 72.4±4.9 |
|  | EC1302 | 13.1±2.6 | 11.3±1.8 | 81.4±16.8 | 52.5±7.1 |
|  | EC1303 | 12.5±0.0 | 10.0±0.0 | 67.1±0.0 | 43.0±0.0 |
| AOA (Superior) | CD | 3.7±0.9 | 2.8±0.4 | 34.2±7.8 | 21.4±2.2 |
|  | Grab5 | 4.4±1.1 | 3.7±0.5 | 51.0±9.6 | 35.7±3.3 |
|  | SteC | 5.3±2.4 | 2.0±0.0 | 50.0±19.2 | 24.4±2.2 |
|  | Grab10 | 6.0±0.4 | 6.0±0.6 | 36.5±12.1 | 24.1±5.9 |
|  | UWM | 6.2±1.5 | 4.8±0.4 | 44.1±13.4 | 23.2±2.5 |
|  | Grab6 | 8.9±3.3 | 6.5±1.6 | 57.9±14.8 | 27.8±5.7 |
|  | Grab9 | 5.6±0.5 | 5.0±0.0 | 29.8±6.2 | 21.7±3.0 |
|  | WM | 5.7±0.4 | 5.8±0.4 | 36.6±7.7 | 27.6±4.4 |
|  |  |  |  |  |  |
| AOB (Erie) | EC1300 | 10.7±1.9 | 9.1±0.9 | 27.9±5.0 | 20.6±2.1 |
|  | EC1301 | 10.4±2.2 | 9.9±1.4 | 52.5±11.3 | 47.8±11.5 |
|  | EC1302 | 9.1±2.7 | 8.2±2.7 | 46.8±28.3 | 35.3±11.8 |
|  | EC1303 | 14.5±0.0 | 7.0±0.0 | 47.5±0.0 | 15.0±0.0 |

**TABLE S12:** OTU’s in the AOA and AOB communities in the sediments of Lake Erie and Lake Superior. The OTU’s were picked per community using different identity cutoffs (85% and 98%), the communities were rarefiyed and alpha diversity was calculated (mean±SD, n=100).

| Identity |  | 85% | | 98% | |
| --- | --- | --- | --- | --- | --- |
| Singletons |  | + | - | + | - |
| AOA (Erie) | EC1300 | 8.8±0.4 | 8.8±0.4 | 59.5±3.1 | 49.9±1.4 |
|  | EC1301 | 12.1±0.8 | 11.7±0.5 | 75.0±2.7 | 65.7±1.6 |
|  | EC1302 | 11.5±1.0 | 10.4±0.7 | 52.1±3.1 | 41.9±2.3 |
|  | EC1303 | 12.0±0.0 | 10.0±0.0 | 56.0±0.0 | 43.0±0.0 |
| AOA (Superior) | CD | 3.5±0.6 | 2.8±0.4 | 25.0±1.8 | 20.1±0.9 |
|  | Grab5 | 4.1±0.8 | 3.7±0.5 | 38.7±2.5 | 32.6±1.4 |
|  | SteC | 3.9±1.0 | 2.0±0.0 | 31.7±2.3 | 22.9±1.0 |
|  | Grab10 | 5.9±0.3 | 5.9±0.4 | 26.2±1.8 | 21.4±1.0 |
|  | UWM | 5.7±0.9 | 4.8±0.4 | 29.6±2.3 | 21.6±1.0 |
|  | Grab6 | 7.0±1.1 | 6.0±0.8 | 32.5±2.5 | 23.2±1.4 |
|  | Grab9 | 5.6±0.5 | 5.0±0.0 | 23.6±1.6 | 20.1±0.8 |
|  | WM | 5.7±0.4 | 5.8±0.4 | 28.6±1.9 | 24.7±1.2 |
|  |  |  |  |  |  |
| AOB (Erie) | EC1300 | 9.9±0.8 | 8.8±0.4 | 22.8±1.4 | 19.1±0.9 |
|  | EC1301 | 9.6±0.8 | 9.4±0.8 | 36.7±3.2 | 34.4±2.7 |
|  | EC1302 | 7.5±1.3 | 7.0±1.1 | 25.7±2.9 | 22.4±2.4 |
|  | EC1303 | 12.0±0.0 | 7.0±0.0 | 28.0±0.0 | 15.0±0.0 |

**TABLE S13:** Shannon index of AOA and AOB communities in the sediments of Lake Erie and Lake Superior. The OTU’s were picked per community using different identity cutoffs (85% and 98%), the communities were rarefiyed and Shannon index was calculated (mean±SD, n=100).

| Identity |  | 85% | | 98% | |
| --- | --- | --- | --- | --- | --- |
| Singletons |  | + | - | + | - |
| AOA (Erie) | EC1300 | 1.84±0.05 | 1.84±0.05 | 4.47±0.07 | 4.37±0.07 |
|  | EC1301 | 2.29±0.05 | 2.29±0.04 | 5.08±0.06 | 4.98±0.06 |
|  | EC1302 | 1.17±0.06 | 1.14±0.06 | 3.23±0.07 | 3.11±0.08 |
|  | EC1303 | 2.08±0.00 | 2.05±0.00 | 4.59±0.00 | 4.44±0.00 |
| AOA (Superior) | CD | 0.69±0.03 | 0.68±0.03 | 2.64±0.07 | 2.56±0.07 |
|  | Grab5 | 0.54±0.04 | 0.52±0.04 | 3.95±0.06 | 3.87±0.05 |
|  | SteC | 0.49±0.03 | 0.46±0.03 | 3.42±0.06 | 3.31±0.05 |
|  | Grab10 | 1.43±0.04 | 1.42±0.04 | 3.05±0.06 | 2.99±0.05 |
|  | UWM | 1.19±0.04 | 1.18±0.03 | 3.22±0.06 | 3.11±0.05 |
|  | Grab6 | 1.06±0.04 | 1.05±0.04 | 3.08±0.06 | 2.95±0.06 |
|  | Grab9 | 1.55±0.04 | 1.53±0.03 | 3.07±0.04 | 3.02±0.04 |
|  | WM | 1.48±0.04 | 1.47±0.04 | 3.09±0.07 | 3.03±0.05 |
|  |  |  |  |  |  |
| AOB (Erie) | EC1300 | 2.34±0.06 | 2.31±0.05 | 3.28±0.07 | 3.18±0.08 |
|  | EC1301 | 1.85±0.11 | 1.88±0.11 | 3.90±0.15 | 3.85±0.14 |
|  | EC1302 | 1.28±0.11 | 1.29±0.10 | 2.61±0.17 | 2.53±0.17 |
|  | EC1303 | 1.84±0.00 | 1.67±0.00 | 3.38±0.00 | 3.02±0.00 |

**References:**

Francis, C.A., K.J. Roberts, J.M. Beman, A.E. Santoro, and B.B. Oakley (2005) Ubiquity and diversity of ammonia-oxidizing archaea in water columns and sediments of the ocean. Proc. Natl. Acad. Sci. 102:14683-14688.

Rotthauwe, J.H. K.-P. Witzel, and W. Liesack (1997) The ammonia monooxygenase structural gene amoA as a functional marker: Molecular fine-scale analysis of natural ammonia-oxidizing populations. Appl. Environ. Microbiol. 63:4704-4712.
